# Effects of Biofertilizer and Green Manure on Soil Bacterial Community in Korla Fragrant Pear Orchard

**DOI:** 10.3390/microorganisms13102252

**Published:** 2025-09-25

**Authors:** Jie Li, Xing Shen, Bolang Chen, Zhanyi He, Linsen Yan, Lele Yang, Bangxin Ding, Zhongping Chai

**Affiliations:** 1 College of Resources and Environment, Xinjiang Agricultural University, No. 311 East Agricultural University Road, Urumqi 830052, China; 18299419152@163.com (J.L.); shenxing@xjau.edu.cn (X.S.); chenwang200910@sina.com (B.C.); hezhanyi666@163.com (Z.H.); ylsen_1946@163.com (L.Y.); ylle_1988@163.com (L.Y.); 2 Xinjiang Key Laboratory of Soil and Plant Ecological Processes, Urumqi 830052, China

**Keywords:** Korla fragrant pear orchard, green manure planting density, biofertilizer, soil bacterial community, soil ecological health, sustainable orchard management

## Abstract

The sustainability of Korla fragrant pear orchards has been increasingly threatened by prolonged intensive agricultural practices. In response, biofertilizers and green manures have gained attention due to their potential to enhance soil structure, activate microbial functions, and improve nutrient uptake. However, the dynamic changes in soil bacterial communities under such interventions remain inadequately understood. This study was conducted from 2022 to 2023 in 7- to 8-year-old Korla fragrant pear orchards in Bayin’guoleng Mongol Autonomous Prefecture, Xinjiang. The treatments included: conventional fertilization (CK), biofertilizer (JF), oil sunflowers (DK1) with 25 cm row spacing and a seeding rate of 27 kg·hm^−2^, oil sunflowers (DK2) with 25 cm row spacing and a seeding rate of 33 kg·hm^−2^, sweet clover (CM1) with 20 cm row spacing and a seeding rate of 21 kg·hm^−2^, and sweet clover (CM2) with 20 cm row spacing and a seeding rate of 27 kg·hm^−2^. During the 2023 pear season, soil samples from the 0–20 cm layer were collected at the fruit setting, expansion, and maturity stages. Their physical and chemical properties were analyzed, and the structure and diversity of the soil bacterial community were examined using 16S rRNA gene high-throughput sequencing. Fruit yield was assessed at the maturity stage. Compared to CK, the relative abundance of *Actinobacteria* increased by 101.00%, 38.99%, and 50.38% in the JF, DK2, and CM1 treatments, respectively. DK1 and CM1 treatments resulted in a 152.28% and 145.70% increase in the relative abundance of the taxon *Subgroup_7*, while JF and DK2 treatments enhanced the relative abundance of the taxon *Gitt-GS-136* by 318.91% and 324.04%, respectively. The Chao1 index for CM2 was 18.76% higher than CK. LEfSe analysis showed that the DK2 and CM2 treatments had a more significant regulatory effect on bacterial community structure. All treatments led to higher fruit numbers and yield compared to CK, with JF showing the largest yield increase. Fertilizer type, soil nutrients, and bacterial community structure all significantly positively influenced pear yield. In conclusion, high-density oil sunflower planting is the most effective approach for maintaining soil microbial community stability, followed by low-density sweet clover. This study provides a systematic evaluation of the dynamic effects of bio-fertilizers and different green manure planting patterns on soil microbial communities in Korla fragrant pear orchards, presenting practical, microbe-based strategies for sustainable orchard management.

## 1. Introduction

Soil microorganisms are recognized as fundamental components of the soil microecosystem. Through the stabilization of rhizosphere microdomains, they directly influence plant nutrient acquisition and drive essential nutrient cycling processes [1]. Among these, soil bacteria play a critical role in improving soil fertility and maintaining ecological balance. Assessing the abundance and spatial distribution of functionally specific bacterial groups is regarded as a key approach to understanding the dynamics of soil ecosystems [2,3]. In traditional agricultural systems, productivity has been largely sustained through the combined use of mineral fertilizers and intensive cropping practices. However, this approach has significantly accelerated soil degradation and contributed to environmental decline [4]. As a result, identifying efficient and environmentally sustainable alternatives to mineral fertilization has become a central focus of agricultural research.

In modern agricultural systems, biofertilizers and green manures, as environmentally friendly fertilization technologies, have demonstrated considerable potential in enhancing soil microbial activity and improving soil health. Previous research has shown that the application of microbial inoculants induces notable shifts in soil microbial communities, influencing microbial activity and plant growth through their specific functional traits or interactions with indigenous microorganisms [5,6]. Chen [7] reported that inoculation with *Bacillus subtilis* altered the relative abundance of dominant soil phyla and increased the effective nitrogen content. The application of *Bacillus subtilis*-enriched biofertilizer was shown by Ma [8] to promote pear seedling growth by enriching functional soil bacterial communities. Green manure intercropping, a sustainable soil management strategy, has been increasingly applied to various crops in recent years [9,10]. Green manures are generally categorized into leguminous and non-leguminous types. Non-leguminous green manures are typically erect with well-developed root systems and possess strong nutrient enrichment and transformation capacities [11]. In contrast, leguminous species fix atmospheric nitrogen into plant-available forms, significantly enhancing ecosystem efficiency, particularly in systems with long-term nitrogen deficiency [12,13]. Zhou [14] conducted a 160-day experiment using three leguminous and three gramineous green manure species and found that soil bacterial diversity was higher under leguminous cover. Xie’s findings revealed that planting green manure in citrus orchards significantly boosts bacterial community diversity and enhances the relative abundance of beneficial bacteria [15]. When green manure is intercropped with other crops and incorporated into the soil, it provides nutrients and organic matter, enhances soil temperature, pH, moisture, and fertility, stimulates microbial activity, increases productivity, decreases the reliance on chemical nitrogen fertilizers, promotes organic matter accumulation, improves nitrogen efficiency, reduces soil erosion, and fosters greater microbial diversity, forming a sustainable nutrient management system [16,17,18].

The Korla fragrant pear (*Pyrus sinkiangensis* Yü) is a unique economic fruit from Xinjiang, designated as a National Geographical Indication Product. Widely cultivated in southern Xinjiang, China, it serves as a major income source for local farmers and herders, while also playing a critical role in regional economic development and rural revitalization. Korla fragrant pear orchards, as typical oasis systems in arid environments, depend heavily on water and soil resources, and their ecological sustainability is threatened by issues such as soil salinization, organic matter depletion, and water stress. Biological fertilizers and green manure, as green fertilization methods, show promise in improving soil microbial activity and ecological balance. Detailed studies on the population abundance of different soil microorganism functional groups are crucial for elucidating the mechanisms by which green fertilization alters soil properties [8,15]. However, in arid environments, the dynamic impacts of various fertilization strategies on soil bacterial communities are not yet fully understood, especially regarding the temporal succession of bacterial functional groups throughout the growing season. Sweet clover (*Melilotus officinalis* (L.) Pall.) and oil sunflower (*Helianthus annuus* Linn.) were used as green manure, while biofertilizer was used as a commercial organic fertilizer, to investigate the effects of various fertilization methods on soil bacterial communities in this study. The goal was to enhance scientific understanding of these effects and support timely, effective adjustments to fertilization guidelines. The objectives of this research are: (1) to evaluate the effects of different fertilization methods on the structure, diversity, and yield of soil bacterial communities; (2) to examine the relationships between soil nutrients, bacterial communities, and yield. These findings provide theoretical support for the role of different green manures and bio-organic fertilizers in Korla pear fragrant orchards’ soil ecology, and contribute to the development of strategies for the targeted regulation of soil microecology and the optimization of fertilization systems in these orchards.

## 2. Materials and Methods

### 2.1. Experimental Site Description

This experiment study was carried out at Heshilik Farm (41°72′78″ N, 85°95′46″ E; elevation: 855.3 masl) in Korla City, Bayin’guoleng Mongol Autonomous Prefecture, Xinjiang, China. The study area lies in central Xinjiang, at the southern foothills of the Tianshan Mountains and the northeastern margin of the Tarim Basin, bordered to the south by the Taklamakan Desert and to the north by Tianshan’s branches. The region experiences a warm temperate continental climate characterized by large diurnal temperature variation, high solar radiation (average annual sunshine: 2990 h), a mean annual temperature of 14–15 °C, annual precipitation of 50–58 mm, and maximum evaporation reaching 2788.2 mm. The effective accumulated temperature ranges from 4100 to 4400 °C, with a frost−free period of 210–239 days. The prevailing wind direction is from the northeast. The soil in the Korla fragrant pear orchard was classified as sandy. Baseline soil nutrient data collected in 2023 indicated a pH of 7.80, organic matter (SOM) of 11.22 g·kg^−1^, available phosphorus (AP) at 12.06 mg·kg^−1^, available potassium (AK) at 167.17 mg·kg^−1^, and alkali-hydrolyzed nitrogen (AN) at 16.63 mg·kg^−1^.

### 2.2. Experimental Design

The experiment was conducted from 2022 to 2023 using 7–8-year-old Korla fragrant pear trees, with Du Pear (*Pyrus betulifolia*) employed as the grafting rootstock. Plants were spaced at 3 m × 5 m, resulting in a density of 675 plants per hectare. In the pear orchard, six treatments were applied: conventional fertilization (CK), biofertilizer (JF), oil sunflower treatment 1 (DK1) with a row spacing of 25 cm and seeding rate of 27 kg·hm^−2^, oil sunflower treatment 2 (DK2) with a row spacing of 20 cm and seeding rate of 33 kg·hm^−2^, sweet clover treatment 1 (CM1) with a row spacing of 25 cm and seeding rate of 21 kg·hm^−2^, and sweet clover treatment 2 (CM2) with a row spacing of 20 cm and seeding rate of 27 kg·hm^−2^. Field trials were conducted with different fertilization treatments during the growth period. The fertilizer application rates, green manure crop types, and seeding techniques are provided in Table 1.

Each treatment was applied to an area of 666.67 m^2^ with three replicates, resulting in a total experimental area of 12,000 m^2^. A one-time basal application of 15,000 kg·hm^−2^ sheep manure was applied in autumn. Calcium superphosphate (46% P_2_O_5_) and potassium sulfate (51% K_2_O) were applied as phosphorus and potash fertilizers, respectively, in a single application in spring before bud break. Urea (46% N) served as the nitrogen fertilizer. The biofertilizer Sprang (2.22% N, 0.05% P_2_O_5_, 3.77% K_2_O, 48.6% organic matter), containing 520 million viable *Bacillus subtilis* per gram, was supplied by Sumisho Fertilizer (Qingdao) Co. Ltd. (Qingdao, China). Sixty percent of the nitrogen and biofertilizer dosage was applied before budding, with the remaining 40% applied during the pre-expansion stage. Fertilizers were evenly distributed in a ring-shaped trench approximately 30 cm deep and wide, dug 50–80 cm from the base of the stem.

The experimental green manure species included sweet clover and oil sunflower, with seeds sourced from the Inner Mongolia Autonomous Region and purchased from Gansu Lambin Ecological Science and Technology Co. in Lanzhou, Gansu Province, China. Seed purity was recorded as 95% for sweet clover and 90% for sunflower, with germination rates of 85% and 80%, respectively. The distance between pear tree rows and green manure rows was controlled at 70–80 cm. Green manure was sown in early April and incorporated into the soil by crushing and burying in late July.

The biofertilizer was selected based on the microbial fertilizers commonly used in the study area. Due to its low nutrient content, fertilizer amounts in treatments receiving biofertilizer were adjusted downward to maintain consistent total nutrient application across all treatments, based on the nutrient content (N, P_2_O_5_, and K_2_O) of the biofertilizer. Nutrient inputs were controlled by the application of chemical fertilizers, with adjustments for the nutrient content of the biofertilizer. The nutrient contribution of green manure biomass was not quantified in this study.

### 2.3. Test Determination Method

#### 2.3.1. Soil Sample Collection and Processing

Soil samples were collected from the pear orchard during the fruit setting (6 June), fruit expansion (3 August), and maturity (6 September) stages in 2023. Using a five-point sampling method, five healthy and similarly vigorous balsam pear trees were selected and tagged per treatment. Soil was collected from the 0–20 cm layer adjacent to both sides of the fertilization trench following removal of surface debris. Samples from both sides were homogenized into one composite sample. The soil was then crushed, mixed, sealed in bags, and transported under cooled conditions with dry ice to the laboratory. Upon return to the laboratory, soil samples were sieved through a 2 mm mesh after removing roots and stones. Each sample was then split into two portions: one was stored at 4 °C for one week for the analysis of soil microbial biomass carbon and nitrogen, as well as microbial community diversity at Parsonage Biotechnology Ltd., Shanghai, China; the other was air-dried, passed through a 1 mm sieve, and used for soil physicochemical analyses. In this study, five soil samples were collected for each treatment, then combined into a single composite sample. To ensure accuracy and repeatability, the composite sample was measured three times in the laboratory.

#### 2.3.2. Soil Physicochemical Analysis

Soil physicochemical properties were determined following the procedures outlined in *Soil Agrochemical Analysis* by Bao [19]. Soil pH was measured using a pH meter with a 2.5:1 water-to-soil ratio, and electrical conductivity (EC) was measured with a conductivity meter. Total nitrogen (TN), soil organic matter (SOM), alkali-hydrolyzed nitrogen (AN), available phosphorus (AP), and available potassium (AK) were analyzed using the semi-micro Kjeldahl method, potassium dichromate external heating method, alkali-hydrolysis diffusion method, sodium bicarbonate extraction-molybdenum-antimony colorimetric method, and ammonium acetate extraction-flame photometric method, respectively.

#### 2.3.3. Soil Microbial Biomass

Soil microbial biomass carbon (MBC) and soil microbial biomass nitrogen (MBN) were determined by the fumigation extraction–volume fraction method and fumigation extraction–ninhydrin colorimetric method [20].

The calculation for MBC is MBC = *Ec*/k_EC_ (1)

where *Ec* represents the difference in organic carbon between fumigated and non-fumigated soil, while k_EC_ denotes the conversion factor, assigned a value of 0.38.

The MBN calculation is MBN = *m*E_min_ − N (2)

where E_min_ − N signifies the total nitrogen difference between fumigated and non-fumigated soil, while *m* represents the conversion factor, assigned a value of 5.0.

#### 2.3.4. Measurement of Soil Microbial Communities

A total of 54 samples were processed in this study, which were collected from the fruit setting, fruit expansion, and maturation stages of Korla fragrant pear. Soil DNA extraction, PCR amplification, and high-throughput sequencing analysis were carried out using previous research methods [21]. The detailed determination method is shown in the annex.

#### 2.3.5. Yield Determination

At the maturity stage of the fragrant pear, five fruits were randomly selected from top to bottom in each of the four cardinal directions (east, south, west, and north) around each sampled tree. The fruits were weighed, and the total number of fruits per treatment was recorded. Yield per plant was calculated using the following formula:

Yield per plant = total number of fruits per plant × average fruit weight

The yield per hectare (1 hm^2^) was then estimated based on the calculated yield per plant.

### 2.4. Statistical Analysis

Excel 2019 was used for initial data organization. Statistical analysis, including one-way analysis of variance (ANOVA) and Duncan’s test at *p* < 0.05, was performed using IBM SPSS Statistics 27 (Statistical Graphics Corp., Princeton, NJ, USA). Graphing and further analyses were completed using R 4.3.2, OriginPro 2024, and the Genescloud platform (https://www.genescloud.cn, accessed on 6 March 2025). The relative abundance and diversity of bacterial communities were analyzed using one-way ANOVA and non-metric multidimensional scaling (NMDS) with the “vegan” package in R (v3.5.1) [22]. Wayne plots were generated using the “VennDiagram” package in R (v3.5.1) [23], and yield histograms were plotted with OriginPro 2024. Partial least squares path modeling (PLS) analyses were conducted using SmartPLS 4.0. For taxonomic descriptions, we uniformly use the general term taxa in the manuscript (text, figures, and captions) when the data include ASVs that cannot be reliably assigned to a specific genus or species. However, when analyses were performed at a defined taxonomic rank (e.g., phylum level), the corresponding specific term (e.g., phyla) was used. LEfSe (LDA Effect Size) analysis was performed on the Genescloud platform, where linear discriminant analysis (LDA) was applied to identify taxa with significant differential effects across groups.

## 3. Results

### 3.1. Soil Bacterial Community Diversity

**Figure 1:**
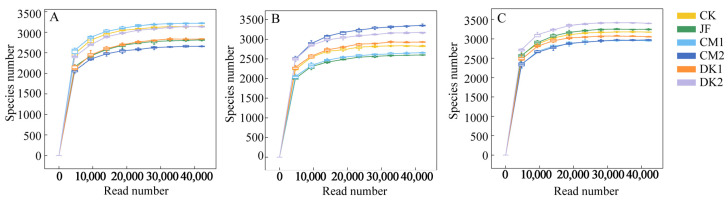
Rarefaction curves of taxa number in different fertilization types. Fruit setting (**A**), fruit expansion (**B**), and maturity (**C**). Conventional fertilization (CK); Biofertilizer (JF); Oilseed sunflower 1 (DK1); Oilseed sunflower 2 (DK2); Sweet clover 1 (CM1); Sweet clover 2 (CM2).

**Figure 2:**
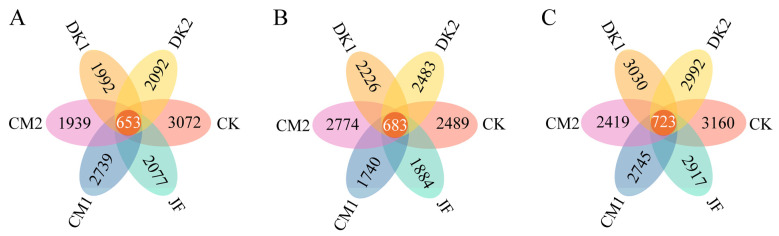
Venn diagram of soil bacterial communities under different fertilization treatments. Fruit setting (**A**), fruit expansion (**B**), and maturity (**C**). Conventional fertilization (CK); Biofertilizer (JF); Oilseed sunflower 1 (DK1); Oilseed sunflower 2 (DK2); Sweet clover 1 (CM1); Sweet clover 2 (CM2).

Rarefaction curves are employed to determine whether sequencing depth is sufficient to represent the microbial community diversity in a sample. As shown in Figure 1, the curves of each sample level off as sequencing depth increases, confirming that the sequencing depth is adequate to capture the sample’s microbial diversity. A Venn diagram was constructed to illustrate the distribution and overlap of ASVs among soil samples and treatment groups. Across all developmental stages—fruit setting, fruit expansion, and maturity—a total of 46,829 bacterial ASVs were detected. At the fruit setting stage (Figure 2A), 14,564 ASVs were detected in the JF, CM1, CM2, DK1, DK2, and CK treatments, with 653 ASVs shared between treatments, accounting for 4.48% of the total. Compared to the CK treatment, the CM1, CM2, DK1, and DK2 treatments showed a reduction of 32.39%, 10.84%, 36.88%, 35.16%, and 31.90% in unique ASVs, respectively. During the fruit expansion stage (Figure 2B), 14,279 bacterial ASVs were detected, with 683 shared across treatments (4.78% of the total). The number of unique ASVs in the CM2 treatment increased by 11.45% compared to CK, while the JF, CM1, DK1, and DK2 treatments had decreases of 24.31%, 30.09%, 10.57%, and 0.24%, respectively. During the maturity stage (Figure 2C), 17,986 ASVs were detected, with 723 shared (4.02% of the total). The number of unique ASVs in the JF, CM1, CM2, DK1, and DK2 treatments decreased by 7.69%, 13.13%, 23.45%, 4.11%, and 5.32%, respectively, compared to CK.

**Figure 3:**
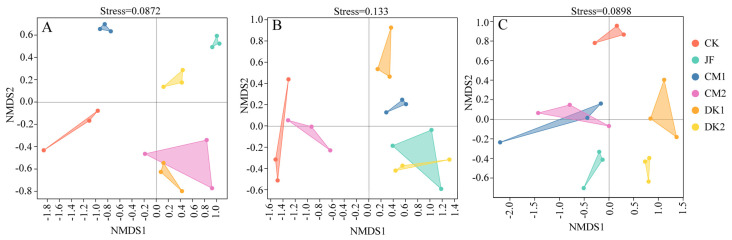
Effects of fertilization type on the *β*-diversity of soil bacterial communities (NMDS analysis). Fruit setting (**A**), fruit expansion (**B**), and maturity (**C**). Conventional fertilization (CK); Biofertilizer (JF); Oilseed sunflower 1 (DK1); Oilseed sunflower 2 (DK2); Sweet clover 1 (CM1); Sweet clover 2 (CM2).

The alpha diversity of soil bacterial communities under different fertilization treatments was analyzed using five standard metrics: Chao1 and Observed species indices (species richness estimates), Shannon and Simpson indices (diversity indices), and Good’s coverage index. These metrics were used to investigate the structural characteristics of microbial communities within each treatment. As presented in Table 2, the bacterial 16S rRNA sequencing library achieved an overall coverage of over 99%, demonstrating that the sequencing data had reached saturation, accurately reflecting the conditions of the samples. During the fruit setting stage (Table 2A), CM1 treatment exhibited the highest values across all indices, with increases of 2.41%, 2.76%, 1.17%, and 0.05%, respectively, relative to CK. The CM1 treatment showed a modest improvement in microbial diversity, though the differences were not significant. Bacterial richness during the fruit expansion stage (Table 2B), as measured by the Chao1 and Observed Species indices, was significantly enhanced under the CM2 treatment, with increases of 18.76% and 15.32% compared to CK; a significant difference in Chao1 was confirmed (*p* < 0.05). The highest Shannon and Simpson diversity indices were found under DK2 treatment, showing increases of 4.22% and 0.42%, respectively, though no statistically significant differences were observed among treatments. These findings indicate a significant effect of CM2 on bacterial richness and a limited, non-significant influence of DK2 on diversity. Bacterial community metrics at the maturity stage (Table 2C)—Chao1, Observed Species, Shannon, and Simpson indices—were highest in the DK2 treatment, with respective increases of 6.90%, 7.36%, 1.76%, and 0.03% compared to CK. Although these values suggest a positive trend, no statistically significant differences were found among treatments. Therefore, DK2 was associated with a modest but non-significant improvement in bacterial richness and diversity. To further investigate microbial community structure, *β*-diversity was calculated using the Bray–Curtis distance and illustrated through NMDS analysis. As depicted in Figure 3, stress values of 0.0872, 0.133, and 0.0898 at the fruit setting, fruit expansion, and maturity stages, respectively, were all below the 0.2 threshold, indicating reliable ordination. Partial overlaps were observed between CM2 and DK1 at the fruit setting stage (Figure 3A), JF and DK2 during the fruit expansion stage (Figure 3B), and CM1 and CM2 at the maturity stage (Figure 3C), while other treatments displayed more distinct separation.

### 3.2. Analysis of the Taxonomic Composition of Soil Bacteria at the Phylum Level

**Figure 4:**
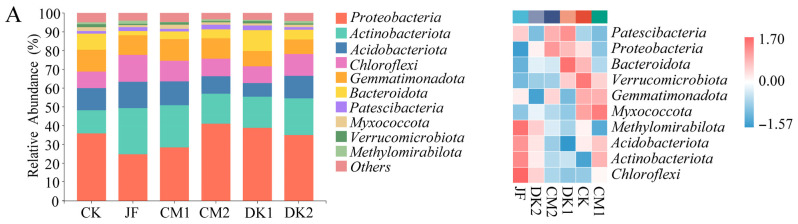

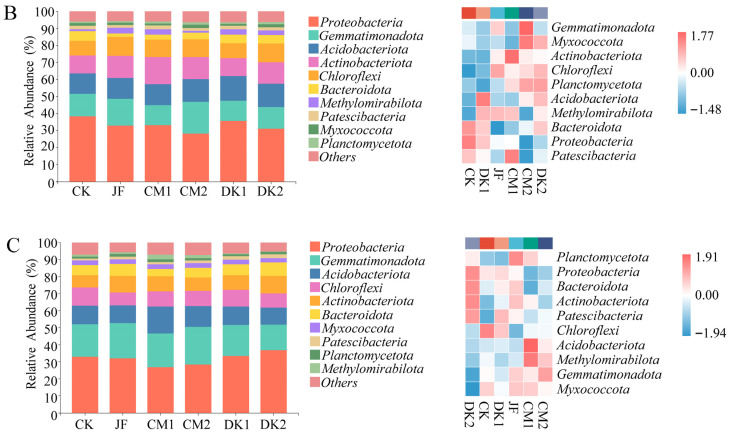
Effects of fertilization type on relative abundance (%) of bacterial communities at phylum level. Fruit setting (**A**), fruit expansion (**B**), and maturity (**C**). Conventional fertilization (CK); Biofertilizer (JF); Oilseed sunflower 1 (DK1); Oilseed sunflower 2 (DK2); Sweet clover 1 (CM1); Sweet clover 2 (CM2).

Figure 4 displays the ten most abundant bacterial phyla in soils treated with biofertilizer and green manure. *Proteobacteria* consistently dominated across all stages, with relative abundance between 31.7% and 33.7%. This was followed by *Actinobacteriota* (8.8–18.6%), *Acidobacteriota* (11.3–13.0%), *Chloroflexi* (9.0–10.6%), *Gemmatimonadota* (10.3–19.1%), *Bacteroidota* (5.9–6.2%), *Myxococcota* (1.5–2.7%), *Patescibacteria* (1.7–1.8%), and *Methylomirabilota* (0.9–2.5%). The remaining phyla maintained relative abundances below 1%. These results suggest that bacterial composition was influenced by treatment and fertility progression.

The application of biofertilizer and green manure at the fruit setting stage (Figure 4A) led to increased *Actinobacteria* abundance across treatments relative to CK. The JF treatment demonstrated a statistically significant increase (*p* < 0.01), with a 101.00% rise compared to CK. Differences among the other treatments were not statistically significant. This suggests that while all treatments showed some enhancement of *Actinobacteria*, the effect was most pronounced under the JF treatment. The relative abundance of the *Gemmatimonadota* phylum decreased to varying degrees across treatments compared with the CK group. However, no statistically significant differences were observed. These results suggest that the application of biofertilizer and green manure may exert a weak inhibitory effect on *Gemmatimonadota* abundance, though the effect was not significant. Across all treatments, the relative abundances of *Proteobacteria*, *Acidobacteriota*, *Chloroflexi*, *Bacteroidota*, *Myxococcota*, *Patescibacteria*, and *Methylomirabilota* fluctuated compared with CK, but without any clear pattern or statistical significance. This indicates that biofertilizer application and green manure planting did not exert a significant influence on the composition of these bacterial groups. In the expansion stage (Figure 4B), treatments with biofertilizer and green manure showed increased relative abundances of *Acidobacteriota* and *Chloroflexi* compared to CK. However, no significant differences were observed. This indicates a non-significant promotive trend of these treatments on the two bacterial phyla. Significant reductions in the relative abundances of *Proteobacteria* and *Bacteroidota* were recorded under CM2 and JF treatments, respectively. *Proteobacteria* decreased by 27.15% in CM2 compared to CK (*p* < 0.05), while *Bacteroidota* decreased by 64.40% in JF compared to CK (*p* < 0.05). These results indicate strong inhibitory effects of CM2 on *Proteobacteria* and of JF on *Bacteroidota*. The application of biofertilizer and green manure resulted in irregular changes in the relative abundances of *Actinobacteriota*, *Gemmatimonadota*, *Myxococcota*, *Patescibacteria*, and *Methylomirabilota* compared to CK. A statistically significant increase (*p* < 0.05) in *Actinobacteriota* was detected under the CM1 treatment, with a 50.38% rise relative to CK, indicating a pronounced promotive effect. In contrast, no significant differences were found for the other phyla, suggesting minimal influence from the treatments. At maturity (Figure 4C), treatments with biofertilizer and green manure led to increases in the relative abundances of *Actinobacteriota* and *Patescibacteria* compared to CK. *Actinobacteriota* showed a significant increase under the DK2 treatment, rising by 38.99% (*p* < 0.05), while changes in *Patescibacteria* were not statistically significant. These results indicate that DK2 significantly enhanced *Actinobacteriota*, while effects on *Patescibacteria* were limited. The application of biofertilizer and green manure resulted in a reduction in *Chloroflexi* abundance compared to CK; however, the changes were not statistically significant, indicating a non-significant inhibitory effect. The relative abundances of *Proteobacteria*, *Acidobacteriota*, *Gemmatimonadota*, *Bacteroidota*, *Myxococcota*, and *Methylomirabilota* showed irregular changes among treatments, with no significant differences detected. Thus, no notable impact of the treatments on these taxa was observed.

### 3.3. Analysis of the Taxonomic Composition of Soil Bacteria Taxa

**Figure 5:**
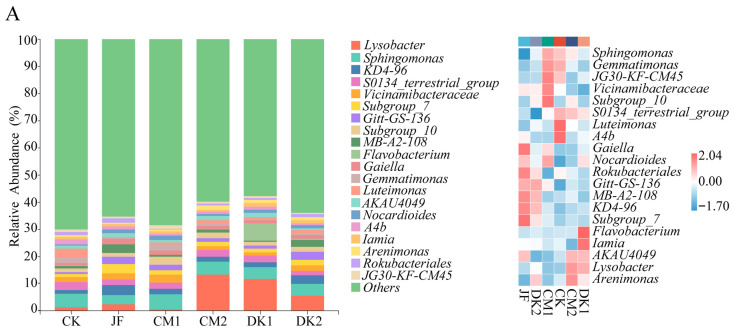

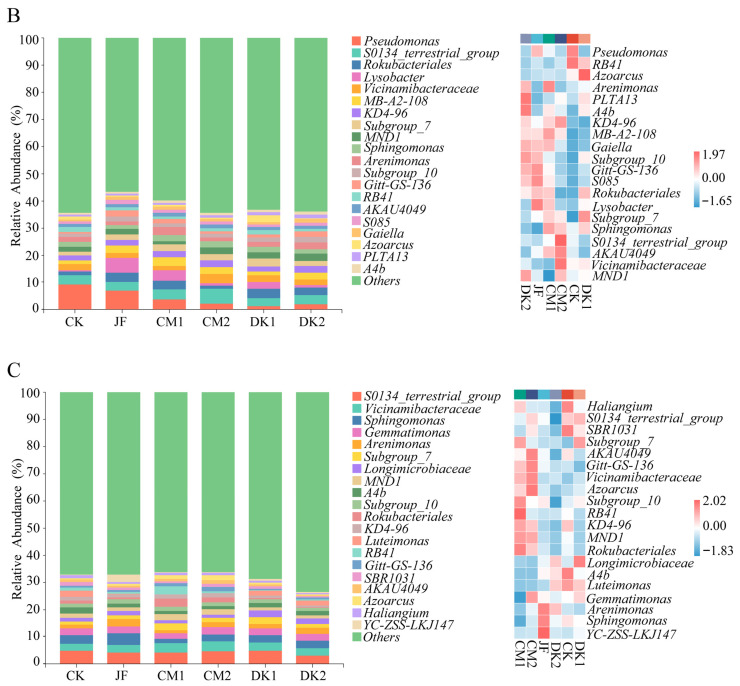
Effects of fertilization type on the relative abundance (%) of bacterial taxa. Fruit setting (**A**), fruit expansion (**B**), and maturity (**C**). Conventional fertilization (CK); Biofertilizer (JF); Oilseed sunflower 1 (DK1); Oilseed sunflower 2 (DK2); Sweet clover 1 (CM1); Sweet clover 2 (CM2).

The top twenty bacterial taxa with the highest relative abundances were selected to assess changes in the soil bacterial community composition during the reproductive stages of pear orchards under different organic fertilizer treatments (Figure 5). The results indicated that dominant bacterial taxa varied across growth stages. However, several taxa consistently maintained a relative abundance above 1% across fruiting, expansion, and maturity stages, including *Sphingomonas* (2.0–4.5%), *KD4-96* (1.2–2.5%), *S0134_terrestrial_group* (2.2–4.3%), *Vicinamibacteraceae* (2.0–3.0%), *Subgroup_7* (1.7–2.1%), *Gitt-GS-136* (0.9–2.1%), *Subgroup_10* (1.4–1.9%), *AKAU4049* (0.8–1.2%), *A4b* (0.8–1.5%), *Arenimonas* (1.0–1.9%), and *Rokubacteriales* (0.9–2.4%). Other taxa did not maintain a relative abundance above 1% throughout all stages, possibly due to treatment-induced shifts in community composition over time.

At the fruit setting stage (Figure 5A), increases in the relative abundances of KD4-96, Subgroup_10, and Gitt-GS-136 were observed under treatments involving biofertilizer and green manure, compared with CK. A significant increase in Gitt-GS-136 was detected in the JF and DK2 treatments, with respective increases of 318.91% and 324.04% (*p* < 0.05). The differences for KD4-96 and Subgroup_10 were not statistically significant, suggesting that although their abundances were elevated, the effects of the treatments were not conclusive. A decline in the relative abundances of *S0134_terrestrial_group* and *A4b* was observed under biofertilizer and green manure treatments compared to CK, although no statistically significant differences were detected. This implies a non-significant inhibitory influence. For *Sphingomonas*, *Vicinamibacteraceae*, *Subgroup_7*, *AKAU4049*, *Arenimonas*, and *Rokubacteriales*, treatment-induced changes in relative abundance were inconsistent and statistically insignificant, indicating no clear impact of the treatments on these taxa. During the fruit expansion stage (Figure 5B), the relative abundances of *Subgroup_7*, *Gitt-GS-136*, *Subgroup_10*, *A4b*, and *Rokubacteriales* increased to varying degrees under biofertilizer application and green manure treatments compared with the CK. Among them, a highly significant increase in *Subgroup_7* was observed in the DK1 treatment, with a 152.28% rise compared to CK (*p* < 0.01). No statistically significant differences were detected for the other taxa across treatments. These findings suggest that while biofertilizer and green manure had a general promoting effect on *Subgroup_7*, the effect was most pronounced under the DK1 treatment. Inconsistent increases and decreases in the relative abundances of Sphingomonas, KD4-96, S0134_terrestrial_group, Vicinamibacteraceae, AKAU4049, A4b, and Arenimonas were observed under biofertilizer and green manure treatments compared to CK. However, none of the changes were statistically significant, suggesting that these treatments did not significantly influence the abundance of these bacterial taxa. At the maturity stage (Figure 5C), the relative abundances of *Vicinamibacteraceae* and *Subgroup_7* increased to varying degrees under biofertilizer application and green manure treatments compared to the CK. A significant increase in *Subgroup_7* was observed in the CM1 treatment, with a 145.70% rise over CK (*p* < 0.05), while no significant differences were found in the other treatments. These findings indicate that *Subgroup_7* responded positively to the treatments, with the most pronounced effect observed under CM1. The relative abundance of *A4b* decreased to varying degrees across treatments compared to the CK, although no statistically significant differences were observed. This suggests that biofertilizer application and green manure planting exerted a mild inhibitory effect on *A4b*, though the effect was not significant. The relative abundances of *Sphingomonas*, *KD4-96*, *S0134_terrestrial_group*, *Gitt-GS-136*, *Subgroup_10*, *AKAU4049*, *Arenimonas*, and *Rokubacteriales* also fluctuated across treatments, but without consistent trends or significant differences. These results indicate that biofertilizer and green manure had no significant impact on these taxa.

### 3.4. LEfSe Hierarchical Tree and LDA Discriminant Results of Soil Bacteria Taxa

**Figure 6:**
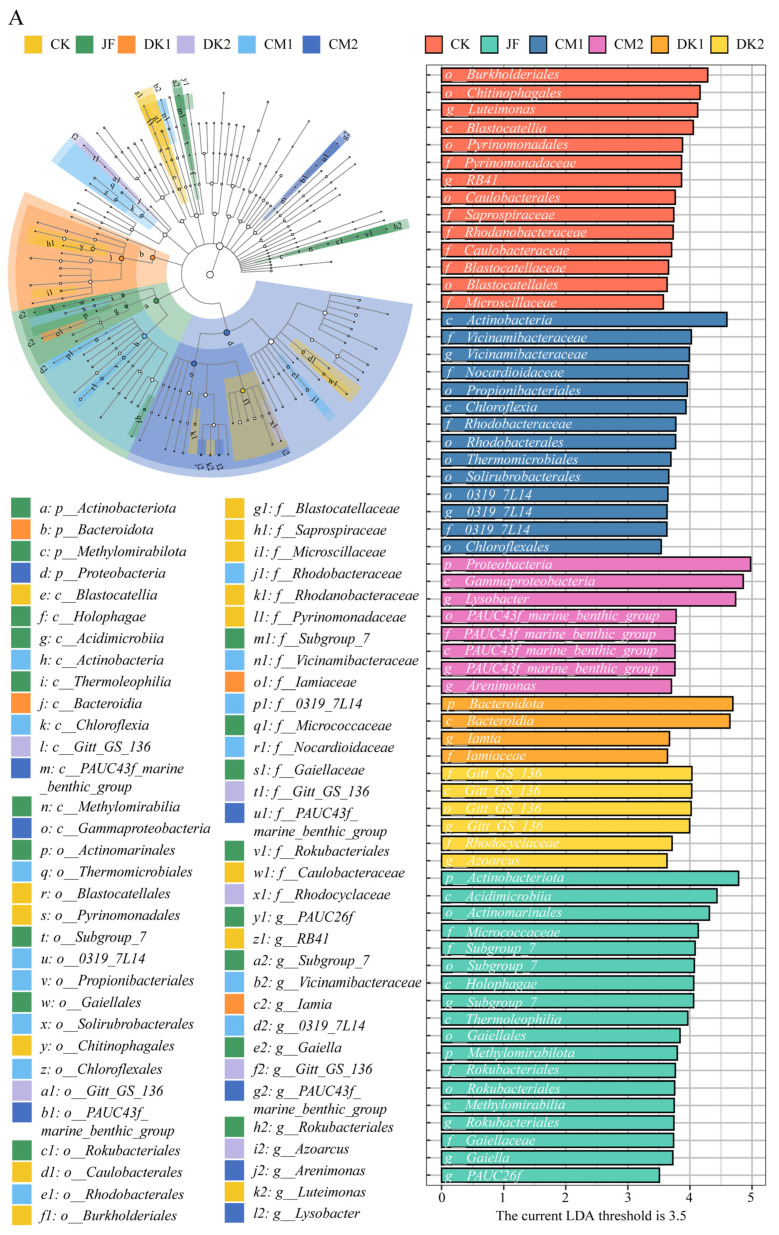

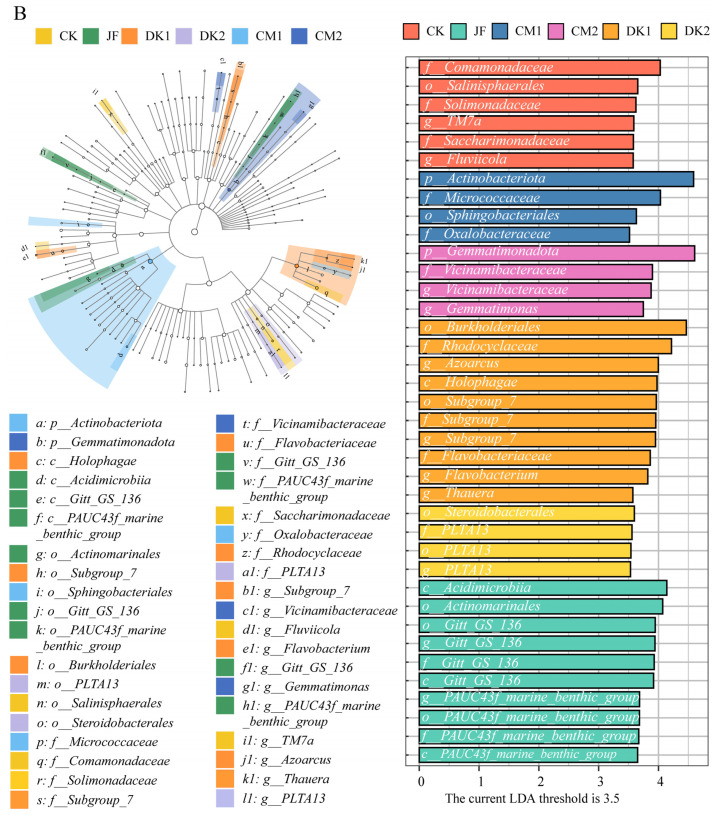

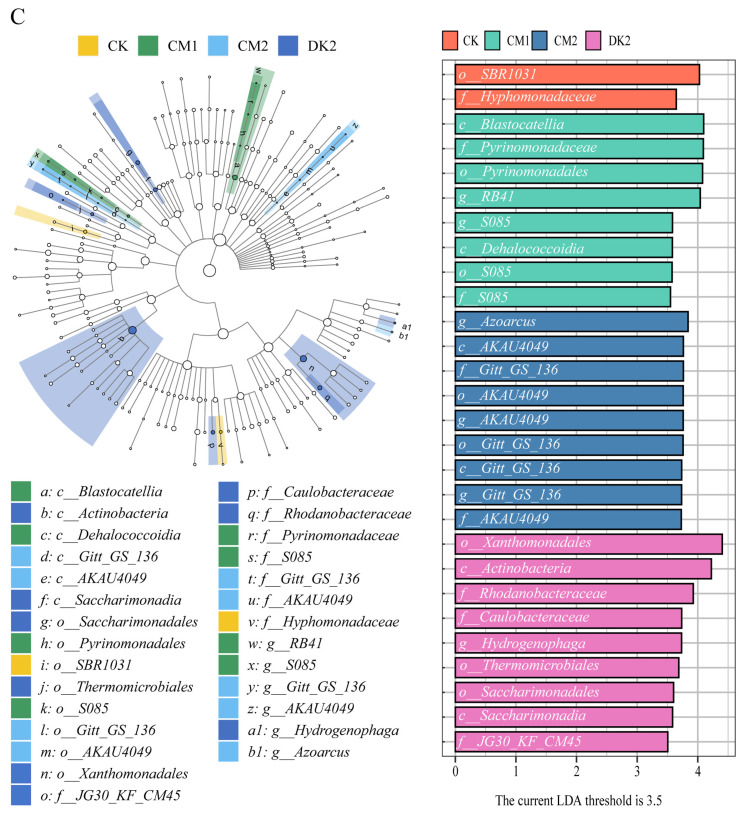
Effects of fertilization type on LEfSe multi-level differential taxa. Fruit setting (**A**), fruit expansion (**B**), and maturity (**C**). Conventional fertilization (CK); Biofertilizer (JF); Oilseed sunflower 1 (DK1); Oilseed sunflower 2 (DK2); Sweet clover 1 (CM1); Sweet clover 2 (CM2). Colored nodes indicate bacterial taxa significantly enriched in the corresponding treatment groups and contributing to intergroup differences. Hollow nodes represent taxa without significant differences or without a notable impact on group separation.

To further assess differences in soil bacterial communities under different fertilization treatments across growth stages, linear discriminant analysis effect size (LEfSe; LDA > 3.5, *p* < 0.05) was used to identify taxa with significant variation at multiple taxonomic levels (Figure 6). During the fruit setting stage (Figure 6A), 64 differential taxa were identified across treatments. CK treatment showed 14 enriched taxa, with *Burkholderiales* (order), *Luteimonas* (genus), and *Chitinophagales* (order) the most prominent. In the JF treatment (18 taxa), the most discriminative were *Actinobacteriota* (phylum), *Acidimicrobiia* (class), and *Actinomarinales* (order). CM1 showed 14 taxa, including *Actinobacteria* (class), *Vicinamibacteraceae* (family), and *Vicinamibacteraceae* (genus). CM2 enriched 8 taxa, particularly *Proteobacteria* (phylum), *Gammaproteobacteria* (class), and *Lysobacter* (genus). DK1 had 4 taxa, notably *Bacteroidota* (phylum), *Bacteroidia* (class), and *Iamia* (genus). DK2 had 6 taxa, including *Gitt–GS–136* (family), and *Gitt–GS–136* (class), and *Gitt–GS–136* (order). The highest number of markers was observed in JF, followed by CM1 = CK > CM2 > DK2 > DK1. At the fruit expansion stage (Figure 6B), 38 differential taxa were detected. CK had 6, with *Comamonadaceae* (family), *Salinisphaerales* (order), and *Solimonadaceae* (family) being dominant. JF showed 10, notably *Acidimicrobiia* (class), *Actinomarinales* (order), and *Gitt–GS–136* (order). CM1 (4 taxa) was associated with *Actinobacteriota* (phylum), *Micrococcaceae* (family), and *Sphingobacteriales* (order). CM2 enriched 4 taxa including *Gemmatimonadota* (phylum), and *Vicinamibacteraceae* (family), and *Vicinamibacteraceae* (genus). DK1 (10 taxa) featured *Burkholderiales* (order), *Rhodocyclaceae* (family), and *Azoarcus* (genus). DK2 enriched 4 taxa, such as *Steroidobacterales* (order), *PLTA13* (family), and *PLTA13* (order). JF and DK1 showed the highest marker counts, followed by DK1 = JF > CK > DK2 = CM1 = CM2. At the maturity stage (Figure 6C), 28 significant taxa were identified. CK had 2, including *SBR1031* (order) and *Hyphomonadaceae* (family). No taxa were significantly enriched in JF or DK1. CM1 enriched 8 taxa, including *Blastocatellia* (class), *Pyrinomonadaceae* (family), and *Pyrinomonadales* (order). CM2 enriched 9 taxa, such as *Azoarcus* (genus), *AKAU4049* (class), and *Gitt–GS–136* (order). DK2 had 9 taxa, including *Xanthomonadales* (order), *Rhodanobacteraceae* (family), and *Actinobacteria* (class). The highest marker counts were observed in DK2 and CM2, followed by DK2 = CM2 > CK > JF = CM1 = DK1.

In summary, the soil bacterial community structure in pear orchards varied significantly across fertility stages and fertilization treatments, with distinct differences in both the number and taxonomic levels of affected taxa. During the fruit setting stage, the greatest number of differential taxa was observed in the JF treatment, particularly among *Actinobacteria*-related groups, suggesting a strong regulatory role in community structure. At the fruit expansion stage, DK1 and JF treatments exhibited the most taxonomic divergence. By the maturity stage, the number of differential taxa declined overall, with only CM2 and DK2 treatments retaining a few dominant groups. These findings indicate that the influence of fertilization methods on soil microbial communities was specific to each fertility stage.

### 3.5. Korla Fragrant Pear Yield

**Figure 7:**
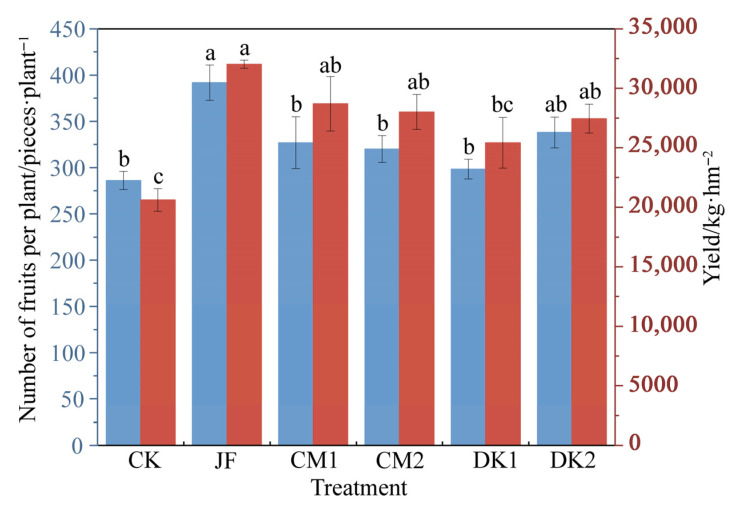
Effects of fertilization type on yield of Korla fragrant pear. Number of fruits per plant (blue); yield (red). Different lower-case letters indicate statistically significant differences between treatments (*p* < 0.05), as determined by one-way ANOVA followed by Duncan’s multiple range test.

As shown in Figure 7, all fertilization treatments significantly increased the yield of Korla fragrant pear compared with the CK treatment. The overall yield performance followed the order JF > CM1 > CM2 > DK2 > DK1 > CK. Yields under JF, CM1, CM2, and DK2 treatments increased by 55.31%, 39.22%, 35.91%, and 33.14%, respectively, compared with CK (*p* < 0.05), suggesting that the application of biofertilizer and the incorporation of green manure were associated with yield improvement, with JF showing the greatest effect. Similarly, the number of fruits per plant increased across all treatments, ranked as JF > DK2 > CM1 > CM2 > DK1 > CK, with a significant 36.95% increase observed in the JF treatment compared to CK (*p* < 0.05). These findings suggest that biofertilizer combined with green manure significantly improves both fruit number and overall yield, with the JF treatment showing the strongest effect.

### 3.6. Regression Analysis of the Relationship Between Type of Fertilizer Application and Microbial, Soil Nutrient and Yield Indicators

**Figure 8:**
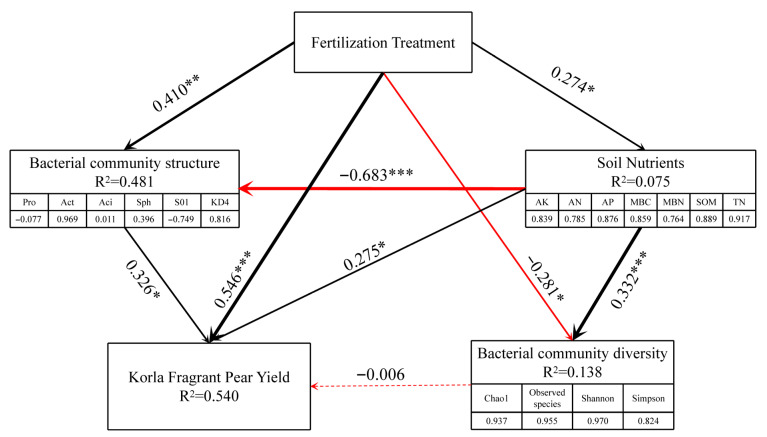
The PLS model was used to analyze the direct and indirect effects of fertilizer types on soil nutrients, soil microbial community, and yield. The width of the arrows represents the magnitude of the standardized path coefficients. The black and red arrows represent positive and negative causal relationships, respectively. * means a significant difference at the *p* < 0.05 level. ** means a significant difference at the *p* < 0.01 level. *** means a significant difference at the *p* < 0.001 level. R^2^ indicates the total explanatory power of all independent variables on the dependent variable. Pro: *Proteobacteria*; Act: *Actinobacteriota*; Aci: *Acidobacteriota*; Sph: *Sphingomonas*; S01: *S0134_terrestrial_group*; KD4: *KD4–96*. AK: available potassium, AN: alkali-hydrolyzed nitrogen, AP: available phosphorus; MBC: microbial biomass carbon; MBN: microbial biomass nitrogen; SOM: organic matter; TN: total nitrogen.

PLS-PM analysis was performed to examine the associations among fertilizer types, soil nutrient status, bacterial community composition, and fragrant pear yield. Corresponding path coefficients and families of indirect pathways are documented in Supplementary Tables S2 and S3. As shown in Figure 8, fertilizer application significantly improved soil nutrient levels (*p* < 0.05), bacterial community structure (*p* < 0.01), and fragrant pear yield (*p* < 0.01), while significantly reducing bacterial diversity (*p* < 0.05). Soil nutrients were positively correlated with both bacterial diversity (*p* < 0.01) and yield (*p* < 0.05) but negatively correlated with community structure (*p* < 0.01). A significant positive correlation was observed between bacterial community structure and yield (*p* < 0.05). However, the effect of bacterial diversity on yield was weak. This systematic analysis clarified the relationships between fertilization types, soil nutrients, and microbial communities, emphasizing the regulatory role of microorganisms in green fertilization strategies. These findings provide a theoretical foundation for enhancing sustainable productivity in orchard ecosystems.

## 4. Discussion

### 4.1. Effects of Fertilization Type on Soil Bacterial Community Diversity

Microbial diversity plays a crucial role in maintaining soil ecosystem stability, and declines in microbial activity are known to reduce soil quality [24]. Bacteria, as responsive indicators of environmental change, are widely used to assess soil health [25,26]. The findings of this study revealed low shared ASV proportions among treatments at all fertility stages, suggesting that fertilization promoted bacterial community divergence via exogenous microbial input or organic amendments. According to alpha diversity analysis, fertilization methods exerted a significant influence on bacterial community diversity regulation, with CM2 notably improving bacterial community richness during the fruit expansion stage. Yang [27] found that planting grasses in banana orchards led to an influx of organic matter, improved soil nutrient status, and fostered the growth of beneficial bacterial communities, thereby enhancing bacterial richness and diversity. Wang [28] suggested that increasing the application rate of green manure results in higher bacterial community richness and diversity. Similar results were observed in this study, confirming that planting high-density jatropha significantly increases soil bacterial richness during the active growth phase of fruit trees. The CM1 and DK2 treatments showed clear benefits in boosting bacterial community diversity and richness during the fruit set and maturity stages, in agreement with Xie [15]. Rich bacterial communities contribute to enhanced soil nutrient cycling, improving soil health, promoting crop growth, strengthening soil structure, and improving plant stress tolerance [29,30,31]. In contrast, Lyu [32] found that the incorporation of green manure significantly reduced bacterial community diversity, which differs from our results. This difference may be due to the combined effects of factors such as geographical location, climatic conditions, plant species, and the duration of intercropping. NMDS analysis further confirmed the impact of different fertilization regimes on the structure of soil bacterial communities. Clear distinctions in community distribution among treatments during the reproductive period suggested that the application of biofertilizer and green manure altered the microbial community assembly by modifying the microenvironment across different developmental stages of fragrant pear. As this study was conducted in a warm temperate zone characterized by arid and water-limited conditions, these environmental constraints may impose unique selective pressures, differing from those in tropical regions, thereby shaping the trajectory of microbial community succession. Long-term, multi-year observations are warranted to elucidate the mechanisms driving temporal shifts in soil bacterial structure and diversity under sustained green manure cultivation. The LefSe analysis showed a decrease in the total number of microbial markers as the growing season advanced, with major differences between treatments concentrated in the *Proteobacteria*, *Bacteroidota*, *Actinobacteriota*, and *Chloroflexi* phyla. The microbial marker count peaked during the fruit-setting stage, with the highest numbers found in the DK2 and CM2 treatments at maturity. This indicates a stronger response of the bacterial community structure to high-density oil sunflower and sweet clover cultivation in these treatments.

### 4.2. Effects of Fertilization Type on the Structure of Soil Bacterial Communities

Soil microorganisms are essential components of terrestrial ecosystems, and the composition and diversity of soil microbial communities are key indicators of their ecological functions [33]. Among these, bacteria serve as sensitive bioindicators of soil conditions and fulfill diverse ecological roles, ranging from pathogenicity to plant growth promotion. It has been demonstrated that different fertilization strategies significantly influence the structure of soil bacterial communities [34,35]. The dominant bacterial phyla identified in this study were *Proteobacteria*, *Actinobacteriota*, *Acidobacteriota*, *Chloroflexi*, *Gemmatimonadota*, *Bacteroidota*, *Myxococcota*, *Patescibacteria*, and *Methylomirabilota*. The dominant phylum identified in this study is *Proteobacteria*. This phylum plays a central role in the decomposition of organic matter and nitrogen fixation. Specific *Proteobacteria* bacteria fix atmospheric nitrogen into a form usable by plants, particularly in nitrogen-deficient soils, which significantly boosts crop growth. Additionally, *Proteobacteria* participate in nutrient cycling through organic matter decomposition, enhancing both soil fertility and crop productivity [36]. Jing [37] reported that *Proteobacteria*, *Chloroflexi*, *Actinobacteria*, Acidobacteria, and Firmicutes were dominant when different green manures were combined with mineral fertilizers in immature red soils. Similarly, Xie [15] observed that the predominant phyla in citrus orchards amended with leguminous green manure were *Proteobacteria*, *Acidobacteriota*, *Actinobacteriota*, and *Bacteroidota*. Deng [38] found that application of Bacillus subtilis led to dominance by *Actinobacteriota*, *Proteobacteria*, *Chloroflexi*, Firmicutes, and *Acidobacteriota*. These findings are consistent with the present study, suggesting that dominant soil bacterial phyla tend to remain stable across different crop species and fertilization regimes. Variations observed may be attributed to site-specific ecological conditions. The *Actinobacteria* phylum is crucial for soil ecology, particularly in the degradation of organic matter, nitrogen cycling, and the maintenance of soil health. Its members, including various saprophytic bacteria, decompose plant and animal residues, releasing nutrients that are available to plants and supporting soil fertility restoration. *Actinobacteria* also play a role in nitrogen fixation, contributing to soil quality and promoting crop productivity. Additionally, their metabolic products can suppress pathogenic bacteria, thus enhancing plant resistance to diseases [39]. The analysis indicated that fertilization treatments had the greatest effect on *Actinobacteria*, with all treatments promoting its relative abundance. The JF, DK2, and CM1 treatments exhibited the most significant increases, suggesting that biofertilizer, high-density oil sunflower cultivation, and low-density sweet clover planting in pear orchards significantly boost *Actinobacteria* abundance. Previous research has shown that applying biofertilizer [40] and cultivating green manure [41,42,43,44] can significantly enhance the relative abundance of *Actinobacteria*, which aligns with the results observed in this study. The soil in the experimental area is weakly alkaline, providing an ideal environment for *Actinobacteria* growth [45]. This increase in *Actinobacteria* abundance may facilitate the mineralization of total carbon [46,47].

In the present study, dominant soil bacterial taxa included *Sphingomonas*, *KD4-96*, *S0134_terrestrial_group*, *Vicinamibacteraceae*, *Subgroup_7*, *Gitt-GS-136*, *Subgroup_10*, *AKAU4049*, *A4b*, *Arenimonas*, and *Rokubacteriales*. Interestingly, Duan [48] found Sphingomonas to be the only overlapping dominant taxon when leguminous green manure was applied in tea plantations. The observed differences in dominant taxa between the studies are likely attributable to variations in crop type, cultivation practices, experimental duration, and soil characteristics. The analysis indicated that all treatments enhanced the relative abundance of the taxon *Subgroup_7*, with the most notable effects observed in the DK1 and CM1 treatments. This suggests that low-density oil sunflower and sweet clover cultivation in pear orchards exert the strongest influence on the abundance of the taxon *Subgroup_7.* During the fruit-setting stage, the JF and DK2 treatments showed the most significant effects on the relative abundance of the taxon *Gitt-GS-136*, highlighting the key role of biofertilizer and high-density oil sunflower cultivation in promoting the abundance of the taxon *Gitt-GS-136* during the vigorous growth phase of fruit trees. This study demonstrated that different green manure intercropping patterns distinctly influenced microbial functional group composition, corroborating findings from similar multi-cropping systems [13]. While the five fertilization schemes improved soil nutrient status and microbial richness, effective field application depends on matching practices to local orchard conditions-specifically soil texture, pH, and baseline fertility. These insights underscore the need for continued long-term investigations into how such practices interact with environmental variables to shape soil microbial dynamics.

### 4.3. Effects of Fertilization Type on the Yield of Korla Fragrant Pear

The results of this study demonstrated that the application of biofertilizer and varied green manure planting modes effectively enhanced both yield and fruit number per plant in Korla fragrant pear orchard, indicating a significant positive effect of biological practices on orchard productivity. Notably, the biofertilizer treatment exerted the most pronounced influence on fruit yield and fruit count per plant. These findings are consistent with those of Wang [49], who reported that biofertilizer significantly improved fruit yield and quality by optimizing the rhizosphere microbial community and enhancing nutrient availability. The yield-enhancing effect of biofertilizer is primarily attributed to the abundance of beneficial microorganisms, such as *Bacillus subtilis*, actinomycetes, and nitrogen-fixing bacteria. These microbes contribute to improving the rhizosphere microecology, promoting nutrient cycling, and activating soil enzyme activities, thereby enhancing root nutrient uptake, which in turn facilitates flower bud differentiation and fruit development [50]. Comparatively, CM1, CM2, DK1, and DK2 treatments also resulted in yield improvements, suggesting that green manure application serves as an effective and sustainable approach to increasing orchard productivity. Fu [51] reported that planting ryegrass in pear orchards significantly increased yields, while Liang [52] demonstrated that green manure application enhanced soil nitrogen levels and water retention in apple orchards on the Loess Plateau, ultimately improving fruit tree productivity. Sweet clover, a leguminous cover crop, is capable of fixing atmospheric nitrogen via rhizobial symbiosis, thereby enriching soil fertility [53]. This likely contributes to its observed effectiveness in increasing fruit counts per plant, particularly under the CM1 treatment where the effect was most pronounced. The DK1 and DK2 treatments resulted in slightly reduced yield and fruit number per plant relative to CM1 and CM2, likely attributable to the limited nitrogen fixation of non-leguminous taxa used. However, carbon sources released during residue decomposition and microbial activation may have played a compensatory role in supporting tree growth [54]. This indicates that the effects of green manures on yield formation vary by species and should be matched to site-specific soil properties and fertilization dynamics.

### 4.4. Correlation Between Fertilization Types and Microorganisms, Soil Nutrients and Yield

Using PLS-PM modeling, this study revealed that fertilizer type modulated fragrant pear yield by altering soil nutrient availability and bacterial community structure. A significant positive effect was observed on soil nutrients and yield-related microbial traits, while microbial diversity was negatively affected. These outcomes corroborate Wang’s findings [49], which highlighted the role of bio-organic fertilizers in improving pear yield via rhizosphere microbial restructuring. Soil nutrients, as a critical intermediate variable, show positive correlations with bacterial community diversity and pear yield but exhibit a negative correlation with community structure. This may indicate that enhanced nutritional conditions favor the dominance of certain functional bacterial communities, leading to structural homogenization. This phenomenon aligns with Beltran-Garcia’s findings [55], which suggested that sufficient nutritional conditions induce community convergence and lessen ecological niche differentiation. Notably, bacterial community structure exerted a direct and significant positive effect on yield, whereas the influence of diversity was weaker. These results suggest that microbial functionality is more critical than diversity. This interpretation is supported by Wang [56], who emphasized the reliance of high-yielding orchards on specific functional microorganisms rather than overall diversity. In conclusion, this study provides valuable theoretical insights into green fertilization strategies for arid region pear orchards, focusing on the microbial regulation mechanisms that drive orchard sustainability. The results emphasize that regulating microbial communities through fertilization is crucial for enhancing orchard productivity. To ensure the sustained improvement of microbial functions and the long-term viability of agricultural ecosystems, green manure methods should be adapted to local environmental conditions, optimizing fertilization cycles and species choices. Long-term green fertilization can improve soil fertility and maintain microbial community stability [57,58], fostering sustainable orchard productivity [59]. In arid environments, green manure is integral to long-term orchard management strategies. By regularly applying suitable species, orchard productivity and microbial health can be enhanced. Given the unique challenges of arid ecosystems, green fertilization strategies not only improve short-term yields but also support the long-term sustainability of orchard production, contributing to the advancement of green agricultural practices.

## 5. Conclusions

This study explored the effects of bio-fertilizer and different green manure planting patterns on the soil microbial community in Korla fragrant pear orchard and their influence on pear yield through a two-year field trial. Results revealed that biofertilizer application (JF), high-density oil sunflower planting (DK2), and low-density sweet clover (CM1) significantly enhanced the relative abundance of *Actinobacteria*. The most significant impact on the abundance of the taxon *Subgroup_7* was observed with low-density oil sunflower (DK1) and sweet clover (CM1), while the greatest promotion of the abundance of the taxon *Gitt-GS-136* occurred with biofertilizer (JF) and high-density oil sunflower planting (DK2). High-density sweet clover (CM2) planting notably increased bacterial community richness, and low-density sweet clover (CM1) with high-density oil sunflower (DK2) offered benefits for both bacterial diversity and richness. The bacterial community structure was most responsive to high-density oil sunflower (DK2) and sweet clover (CM2) planting. Additionally, both microbial fertilizers and green manure cultivation increase pear yields in Korla pear orchards, with biofertilizer (JF) showing the most pronounced effect on yield improvement. Fertilizer type is a key factor that influences soil ecological processes and orchard productivity, with microbial community structure being central to this process. In summary, high-density oil sunflower planting in 7- to 8-year-old Korla fragrant pear orchards is the most effective in maintaining microbial community stability and health, followed by low-density sweet clover. This study provides microbiological evidence to guide soil health management in Korla fragrant pear orchards. To ensure sustainable orchard production, fertilization strategies should be adjusted to local ecological conditions, and green manure planting patterns should be precisely regulated. This not only optimizes soil microbial community structure but also promotes soil health and boosts orchard productivity, offering theoretical support for sustainable orchard management.

## Figures and Tables

**Table 1:** Experimental scheme for different fertilization treatments.

Treatment	Concrete Content	Seeding Rate/kg·hm^−2^	Planting Depth /cm	Row Spacing/cm	Biofertilizer /kg·hm^−2^	Nutrient Level/kg·hm^−2^		
						N	P_2_O_5_	K_2_O
CK	Only chemical fertilizer	0	0	0	0	300	300	150
JF	Biofertilizer	0	0	0	1200	273.64	299.40	254.76
DK1	Oil sunflower treatment 1	27	2–3	25	0	300	300	150
DK2	Oil sunflower treatment 2	33	2–3	20	0	300	300	150
CM1	Sweet clover treatment 1	21	1	25	0	300	300	150
CM2	Sweet clover treatment 2	27	1	20	0	300	300	150

**Table 2:** Effects of fertilization type on the *α*-diversity of soil bacterial communities. Fruit setting (A), fruit expansion (B), and maturity (C). Conventional fertilization (CK); Biofertilizer (JF); Oilseed sunflower 1 (DK1); Oilseed sunflower 2 (DK2); Sweet clover 1 (CM1); Sweet clover 2 (CM2). Different lowercase letters indicate statistically significant differences between treatments (*p* < 0.05), using one-way ANOVA, followed by Duncan’s multiple range test.

	Treatment	Chao1	Observed_Species	Shannon	Simpson	Goods_Coverage
A	CK	3143.99 ± 139.02 a	3008.63 ± 116.10 ab	10.39 ± 0.07 ab	0.99800 ± 0.00052 a	0.99270 ± 0.0009 a
	JF	2815.04 ± 34.71 a	2650.7 ± 26.51 ab	9.90 ± 0.02 ab	0.99700 ± 0.00007 a	0.99240 ± 0.00021 a
	CM1	3219.86 ± 198.59 a	3091.57 ± 171.48 a	10.51 ± 0.08 a	0.99850 ± 0.00008 a	0.99290 ± 0.00111 a
	CM2	2658.96 ± 230.79 a	2530.90 ± 217.95 b	9.49 ± 0.61 b	0.98230 ± 0.01465 a	0.99360 ± 0.00059 a
	DK1	2837.43 ± 140.76 a	2670.90 ± 126.65 ab	9.45 ± 0.15 b	0.98850 ± 0.00233 a	0.9920 ± 0.00058 a
	DK2	3141.88 ± 116.34 a	2948.33 ± 98.32 ab	10.07 ± 0.07 ab	0.99680 ± 0.00012 a	0.99110 ± 0.00043 a
B	CK	2824.02 ± 37.57 bc	2729.23 ± 27.87 ab	9.99 ± 0.09 ab	0.99420 ± 0.00177 a	0.99410 ± 0.00028 a
	JF	2600.77 ± 200.02 c	2473.4 ± 194.27 b	9.44 ± 0.42 b	0.99020 ± 0.00562 a	0.99350 ± 0.00046 ab
	CM1	2657.04 ± 136.84 c	2522.57 ± 116.42 b	9.75 ± 0.12 ab	0.99610 ± 0.00050 a	0.99340 ± 0.00066 ab
	CM2	3353.75 ± 121.33 a	3147.37 ± 127.00 a	10.33 ± 0.17 a	0.99800 ± 0.00045 a	0.99050 ± 0.00026 c
	DK1	2931.38 ± 119.40 abc	2785.67 ± 115.86 ab	10.12 ± 0.09 ab	0.99760 ± 0.00019 a	0.99250 ± 0.00034 b
	DK2	3173.33 ± 147.73 ab	3038.9 ± 127.20 a	10.41 ± 0.08 a	0.99830 ± 0.00006 a	0.99290 ± 0.00073 ab
C	CK	3177.23 ± 220.47 a	3087.17 ± 195.65 a	10.54 ± 0.13 a	0.99860 ± 0.00021 a	0.99390 ± 0.00134 a
	JF	3248.98 ± 162.28 a	3142.7 ± 151.97 a	10.52 ± 0.14 a	0.99820 ± 0.00039 a	0.99300 ± 0.00064 a
	CM1	2949.95 ± 309.60 a	2854.03 ± 297.02 a	10.19 ± 0.31 a	0.99760 ± 0.00077 a	0.99360 ± 0.00084 a
	CM2	2972.12 ± 217.02 a	2854.67 ± 217.56 a	10.30 ± 0.19 a	0.99820 ± 0.00032 a	0.99330 ± 0.00041 a
	DK1	3056.42 ± 15.55 a	2989.6 ± 20.60 a	10.41 ± 0.07 a	0.99830 ± 0.00018 a	0.99450 ± 0.00064 a
	DK2	3396.59 ± 75.33 a	3314.33 ± 65.02 a	10.72 ± 0.02 a	0.99890 ± 0.00002 a	0.99360 ± 0.00058 a

## Data Availability

The original contributions presented in this study are included in the article. The raw metagenomic sequencing data have been deposited in the NCBI SRA database under the accession number PRJNA1311275. Further inquiries can be directed to the corresponding authors.

## Supplementary Materials

The following supporting information can be downloaded at: https://www.mdpi.com/article/10.3390/microorganisms13102252/s1, Table S1: Soil nutrient content during different stages of plant growth. Table S2: Partial least squares path model (PLS-PM) path coefficients. Table S3: Partial least squares path model (PLS-PM) specific indirect effects path coefficients.
