# Supplementary material for: Effects of Biofertilizer and Green Manure on Soil Bacterial Community in Korla Fragrant Pear Orchard
Source: Microorganisms. 2025 Sep 25;13(10):2252. doi: 10.3390/microorganisms13102252 (PMC12565902; doi:10.3390/microorganisms13102252)
Supplement: Supplementary file 1 [file microorganisms-13-02252-s001.zip › microorganisms-3714985-supplementary.pdf]

Supporting Information

Sequencing process:

PCR amplification of the bacterial 16S rRNA genes V3–V4 region was performed using the forward primer 338F (5'-ACTCCTACGGGAGGCAGCA-3') and the reverse primer 806R (5'-GGACTACHVGGGTWTCTAAT-3'). Sample-specific 7-bp barcodes were incorporated into the primers for multiplex sequencing. The PCR components contained 5 μl of buffer (5×), 0.25 μl of Fast pfu DNA Polymerase (5U/μl), 2 μl (2.5 mM) of dNTPs, 1 μl (10 uM) of each Forward and Reverse primer, 1 μl of DNA Template, and 14.75 μl of ddH_2O. Thermal cycling consisted of initial denaturation at 98 °C for 5 min, followed by 25 cycles consisting of denaturation at 98 °C for 30 s, annealing at 53 °C for 30 s, and extension at 72 °C for 45 s, with a final extension of 5 min at 72 °C. PCR amplicons were purified with Vazyme VAHTSTM DNA Clean Beads (Vazyme, Nanjing, China) and quantified using the Quant-iT PicoGreen dsDNA Assay Kit (Invitrogen, Carlsbad, CA, USA). After the individual quantification step, amplicons were pooled in equal amounts, and pair-end 2×250 bp sequencing was performed using the Illlumina NovaSeq platform with NovaSeq 6000 SP Reagent Kit (500 cycles) at Shanghai Personal Biotechnology Co., Ltd (Shanghai, China).

Software and version used:

In order to make the data of each sample comparable, the pumping depth was randomized according to 95% of the lowest sequence volume, in this way, it also minimizes the effect of different sequencing depths.

(1) Microbiome biological information was analyzed using QIIME2 version 2019.4, modified and improved according to the official tutorial (https://docs.qiime2.org/2019.4/tutorials/). Raw sequence data were processed using demux plugin for decoding, cutadapt plugin for primer excision, and then DADA2 plugin was used for data processing such as quality filtering, denoising, splicing and chimera removal. The sequences obtained above were grouped by 100% sequence similarity to generate characteristic sequence ASVs as well as abundance data tables.

(2) Classification of ASVs/OTUs and identification of taxonomic status: By using the Greengenes database, the characteristic sequences of ASVs/OTUs were compared with the reference sequences in the database to obtain the taxonomic information corresponding to each ASV/OTU. ASVs/OTUs with abundance values lower than 0.001% (1 in 100,000) of the total number of sequenced sequences for all samples were removed, and the abundance matrices of the removed rare ASVs/OTUs were used for a subsequent series of analyses. Meanwhile, the identification results of each sample at each taxonomic level were plotted as bar charts using the R software to visually compare the differences in the number of ASV/OTUs and taxonomic status identification results of different samples.

DADA2 sequence denoising:

Analysis software: QIIME2 (2019.4). Analysis steps: first call qiime cutadapt trim-paired to excise primer fragments of sequences and discard sequences that do not match the primers; then call DADA2 via qiime dada2 denoise-paired for quality control, denoising, splicing, and chimera removal. The above steps were analyzed separately for each library. After completing denoising of all libraries, the ASVs feature sequences and ASV tables were merged, and singletons ASVs were removed (i.e., ASVs with a total number of sequences of only 1 in the

whole sample, the default operation). Sequence Length Distribution Statistics The length distribution of high-quality sequences contained in the full sample was counted using R language scripts.

Table S1

Soil nutrient content during different stages of plant growth.

Period	Treatment	CK	JF	CM1	CM2	DK1	DK2
Fruit setting stage	SOM	10.61±0.16a	11.19±0.15a	11.29±0.43a	11.26±0.26a	11.49±0.42a	11.47±0.22a
	AK	154.33±1.76c	168.00±0.58b	174.33±1.86a	166.33±0.88b	172.67±0.67a	167.33±1.33b
	AP	10.95±0.55a	11.89±0.41a	12.58±0.33a	12.34±0.36a	12.53±0.65a	12.06±0.19a
	AN	15.75±0.29b	16.53±0.31ab	17.00±0.14a	16.57±0.32a	17.07±0.09a	16.88±0.19a
	TN	0.63±0.02c	0.68±0.01bc	0.76±0.01a	0.72±0.02ab	0.72±0.02ab	0.70±0.02b
	MBC	294.37±4.34a	293.89±2.36a	313.30±12.38a	304.73±5.29a	302.98±4.91a	297.61±4.84a
	MBN	25.92±0.44a	28.12±1.59a	29.88±0.35a	28.96±0.27a	29.16±3.84a	28.54±2.49a
Fruit expansion stage	SOM	10.94±0.21c	11.79±0.18b	11.91±0.21b	11.64±0.09b	12.79±0.23a	12.02±0.20b
	AK	160.00±3.06c	172.00±1.53ab	176.67±2.33ab	169.00±1.53bc	181.33±1.20a	168.67±6.33bc
	AP	11.04±0.18c	12.13±0.07b	13.07±0.11a	13.01±0.27a	13.22±0.44a	13.19±0.34a
	AN	15.87±1.69a	17.58±0.58a	17.98±0.15a	17.45±0.59a	17.65±0.15a	17.42±0.08a
	TN	0.67±0.01e	0.73±0.02d	0.88±0.01a	0.84±0.02ab	0.81±0.01bc	0.78±0.02c
	MBC	302.03±9.60b	312.25±4.02ab	332.03±5.23a	325.08±3.17ab	322.13±8.37ab	316.92±8.56ab
	MBN	27.63±0.39d	30.66±0.7c	34.06±0.1a	32.65±0.6abc	33.46±1.07ab	31.86±0.79bc
Maturity stage	SOM	10.87±0.30c	12.17±0.22b	12.55±0.03b	12.26±0.11b	13.51±0.14a	12.76±0.31b
	AK	161.33±4.33c	178.00±1.53b	188.00±1.15a	176.67±4.63b	189.67±1.86a	171.33±3.33b
	AP	11.11±0.14c	12.90±0.20b	14.04±0.17a	13.79±0.24a	13.88±0.15a	13.74±0.08a
	AN	16.25±0.25e	17.13±0.21d	20.00±0.23a	19.05±0.21b	18.80±0.10b	17.88±0.20c
	TN	0.70±0.01d	0.77±0.02c	0.94±0.01a	0.89±0.02b	0.88±0.02b	0.85±0.03b
	MBC	307.51±0.96e	323.27±1.14d	347.80±0.95a	339.06±3.44b	332.58±3.52bc	327.16±2.67cd
	MBN	28.15±0.52c	32.14±0.14bc	37.22±0.18a	36.32±0.43ab	35.94±0.13ab	35.06±3.46ab

Note: SOM: soil organic matter; AK: available potassium; AP: available phosphorous; AN: alkali-hydrolyzed nitrogen; TN: total nitrogen; MBC: microbial biomass carbon; MBN: microbial biomass nitrogen.

Table S2

Partial least squares path model (PLS-PM) path coefficients.

Path	Original sample (O)	Sample mean (M)	STDEV	T statistics (|O/STDEV|)	P values	Significant or not
Bacterial community diversity->Korla Fragrant Pear Yield	-0.006	-0.002	0.096	0.067	0.947	No
Fertilization Treatment->Bacterial community diversity	-0.281	-0.286	0.123	2.293	0.022	Yes
Fertilization Treatment->Korla Fragrant Pear Yield	0.546	0.534	0.134	4.076	0.000	Yes
Fertilization Treatment->Soil Nutrients	0.274	0.274	0.139	1.978	0.048	Yes
Fertilization Treatment->Bacterial community structure	0.41	0.409	0.153	2.687	0.007	Yes
Soil Nutrients->Bacterial community diversity	0.332	0.349	0.084	3.935	0.000	Yes
Soil Nutrients->Korla Fragrant Pear Yield	0.275	0.268	0.132	2.082	0.037	Yes
Soil Nutrients-> Bacterial community structure	-0.683	-0.676	0.091	7.483	0.000	Yes
Bacterial community structure->Korla Fragrant Pear Yield	0.326	0.322	0.141	2.317	0.021	Yes

Table S3

Partial least squares path model (PLS-PM) specific indirect effects path coefficients

Specific indirect effects path	Original sample (O)	Sample mean (M)	STDEV	T statistics (|O/STDEV|)	P values	Significant or not
Soil Nutrients-> Bacterial community structure-> Korla Fragrant Pear Yield	-0.222	-0.218	0.103	2.157	0.031	Yes
Fertilization Treatment-> Soil Nutrients-> Bacterial community structure	-0.187	-0.186	0.102	1.837	0.066	No
Fertilization Treatment-> Bacterial community diversity->Korla Fragrant Pear Yield	0.002	0.002	0.030	0.060	0.952	No
Soil Nutrients-> Bacterial community diversity-> Korla Fragrant Pear Yield	-0.002	0.000	0.036	0.060	0.952	No
Fertilization Treatment-> Soil Nutrients->Bacterial community diversity->Korla Fragrant Pear Yield	-0.001	0.000	0.011	0.054	0.957	No
Fertilization Treatment->Soil Nutrients-> Bacterial community diversity	0.091	0.096	0.056	1.620	0.105	No
Fertilization Treatment-> Bacterial community structure->Korla Fragrant Pear Yield	0.133	0.139	0.085	1.578	0.115	No
Fertilization Treatment->Soil Nutrients-> Korla Fragrant Pear Yield	0.075	0.073	0.054	1.399	0.162	No
Fertilization Treatment->Soil Nutrients-> Bacterial community structure->Korla Fragrant Pear Yield	-0.061	-0.059	0.044	1.400	0.162	No
